# Parallel Synthesis of 2-Substituted 6-(5-Oxo-1-phenylpyrrolidin-3-yl)pyrimidine-5-carboxamides

**DOI:** 10.3390/molecules17055363

**Published:** 2012-05-08

**Authors:** Bojana Črček, Jernej Baškovč, Uroš Grošelj, Drago Kočar, Georg Dahmann, Branko Stanovnik, Jurij Svete

**Affiliations:** 1 Faculty of Chemistry and Chemical Technology, University of Ljubljana, Aškerčeva 5, P.O. Box 537, 1000 Ljubljana, Slovenia; 2 Boehringer Ingelheim Pharma GmbH & Co. KG, Medicinal Chemistry, 88397 Biberach, Germany

**Keywords:** parallel synthesis, pyrimidines, 2-(heteroaryl)ethylamines

## Abstract

A library of 24 6-(5-oxo-1-phenylpyrrolidin-3-yl)pyrimidine-5-carboxamides **10**{*1*,*2*; *1–12*} was prepared by a parallel solution-phase approach. The synthesis comprises a five-step transformation of itaconic acid (**11**) into 1-methyl and 1-phenyl substituted 6-(5-oxo-1-phenylpyrrolidin-3-yl)pyrimidine-5-carboxylic acids **17**{*1*,*2*} followed by parallel amidation of **17**{*1*,*2*} with a series of 12 aliphatic amines **18**{*1–12*} to afford the corresponding carboxamides **10** in good overall yields and in 80–100% purity.

## 1. Introduction

2-[(Hetero)aryl]ethylamines, such as dopamine, histamine, tryptamine, serotonin, and melatonin are representative chemical messengers playing a crucial role in biological processes [1]. Therefore, the preparation of libraries of their novel synthetic analogues is of particular interest and represents an important target in medicinal [2,3,4,5], synthetic organic, and combinatorial chemistry [6,7,8,9,10].

**Figure 1:**
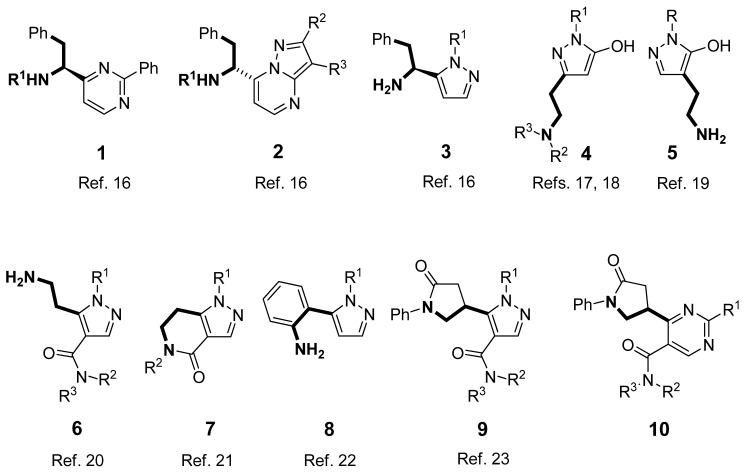
Aminoethyl substituted heterocycles **1**–**10**.

In the last two decades, alkyl 2-substituted 3-(dimethylamino)prop-2-enoates and related enaminones have proven to be easily available and versatile reagents for the preparation of various functionalized heterocycles [11,12,13,14,15]. Recently, a part of our research in this field has been focused on the synthesis of aminoethyl functionalized heterocycles. In this context, we first reported the synthesis of non-racemic 1-heteroaryl-2-phenylethylamines **1**–**3** from α-amino acid derived enaminoketones [16]. Further, the syntheses of the pyrazole analogues of histamine were developed: 2-aminoethyl substituted 1*H*-pyrazole derivatives **4**–**6** as the open-chain analogues [17,18,19,20] and 6,7-dihydro-1*H*-pyrazolo[4,3-*c*]pyridin-4(5*H*)-one derivatives **7** [21], 5-(2-aminophenyl)pyrazole derivatives **8** [22], and 5-(5-oxo-1-phenylpyrrolidin-3-yl)-1*H*-pyrazole-4-carboxamides **9** [23] as the conformationally constrained analogues of histamine. In continuation, we have focused our attention on 2-substituted 6-(5-oxo-1-phenylpyrrolidin-3-yl)pyrimidine-5-carboxamides **10** (Figure 1).

Herein, we report a parallel solution-phase synthesis of a library of 24 6-(5-oxo-1-phenylpyrrolidin-3-yl)pyrimidine-5-carboxamides **10**{*1*,*2*; *1–12*} as novel 2-heteroarylethylamine derivatives.

## 2. Results and Discussion

### 2.1. Synthesis of Title Compounds ***10***

**Scheme 1:**
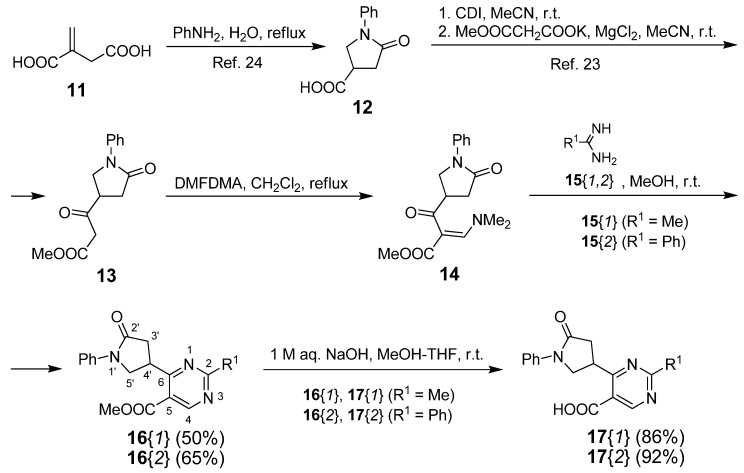
Synthesis of 4-(5-Oxo-1-phenylpyrrolidin-3-yl)pyrimidine-5-carboxylic Acids **17**{*1*,*2*}.

First, the starting compound **12** was prepared from commercially available itaconic acid (**11**) and aniline following the literature procedure [24]. Transformation of **12** into the enaminone **14** as the first key intermediate was performed following the literature protocol [23]: Masamune-Claisen condensation of **12** with 1,1'-carbonyldiimidazole (CDI) as activating agent in anhydrous acetonitrile at room temperature gave the *β*-keto ester **13**, which when treated with *N*,*N*-dimethylformamide dimethylacetal (DMFDMA) in refluxing toluene gave the enaminone intermediate **14**. Subsequent cyclisation of **14** with acetamidine **15**{*1*} and benzamidine **15**{*2*} afforded methyl 4-(5-oxo-1-phenylpyrrolidin-3-yl)pyrimidine-5-carboxylates **16**{*1*} and **16**{*2*} in 50% and 65% yield, respectively. Finally, hydrolysis of **16**{*1*} and **16**{*2*} with 1 M aqueous NaOH in a mixture of methanol and THF at room temperature furnished the corresponding carboxylic acids **17**{*1*} and **17**{*2*} in 86% and 92% yield, respectively (Scheme 1).

**Scheme 2:**
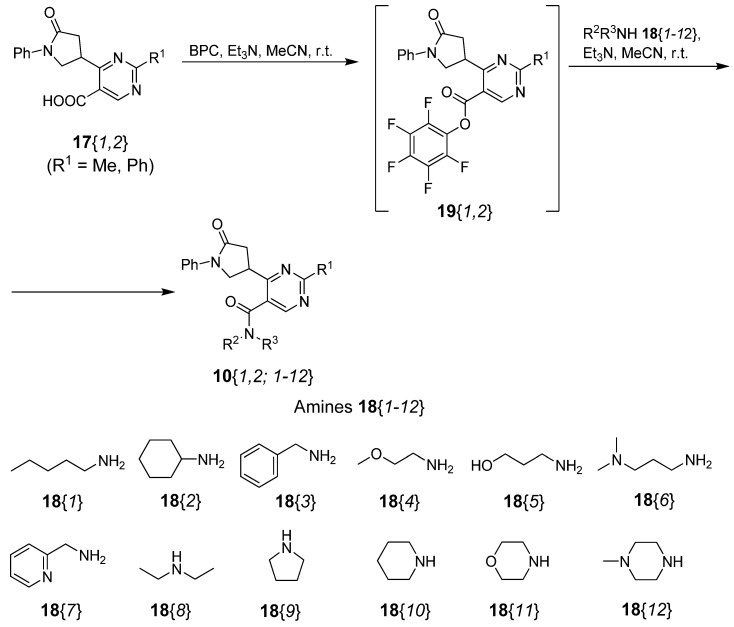
Parallel synthesis of 6-(5-oxo-1-phenylpyrrolidin-3-yl)pyrimidine-5-carboxamides **10**.

With the desired carboxylic acids **17**{*1*,*2*} as the key-intermediates in our hands, a parallel solution-phase synthesis of 2-substituted 6-(5-oxo-1-phenylpyrrolidin-3-yl)pyrimidine-5-carboxamides **10** was studied. We supposed that the reaction conditions for the parallel amidation step as well as the workup protocol should be similar to those employed in the synthesis of closely related pyrazole analogues **9** [23]. Accordingly, bis(pentafluorophenyl) carbonate BPC was chosen as the reagent for activation of the carboxylic acids **17** and acetonitrile as the solvent. Preliminary amidations of **17**{*1*,*2*} with benzylamine (**18**{*3*}) as the model primary amine proceeded smoothly to furnish the desired *N*-benzylcarboxamides **17**{*1*; *3*} and **17**{*2*; *3*}, which precipitated from the reaction mixtures and were isolated by filtration. Somewhat surprisingly, analogous amidations of **17**{*1*,*2*} with diethylamine (**18**{*8*}) did not proceed to completion unless excess diethylamine (**18**{*8*}) was employed. The corresponding carboxamides **17**{*1*; *8*} and **17**{*2*; *8*} did not precipitate from the reaction mixtures and were isolated by evaporation of the reaction mixtures followed by purification by dry flash column chromatography (DFCC) [25,26] over aluminium oxide [27]. Consequently, the following procedure for parallel amidation was applied: the acids **17**{*1*,*2*} were activated with triethylamine and bis(pentafluorophenyl) carbonate (BPC) in acetonitrile at room temperature to give the intermediate pentafluorophenyl esters **19**{*1*,*2*}, which were subsequently treated with 1 equiv. of primary amines **18**{*1–7*} or with 10 equiv. of secondary amines **18**{*8*–*12*} at room temperature for 12 h. Nine products that precipitated from the reaction mixtures were isolated by filtration to afford carboxamides **10**{*1*; *3*,*4*} and **10**{*2*; *1*–*7*} in 28–100% yields and in 84–100% purity (Workup A). The rest of the products, which did not precipitate from the reaction mixtures, were isolated by evaporation of the reaction mixtures followed by purification of the residues by DFCC over aluminium oxide, and evaporation of the eluates to give compounds **10**{*1*; *1*,*2*,*5*–*12*} and **10**{*2*; *8*–*12*} in 40–100% yields and in 80–100% purity (Workup B). In this manner, all 24 carboxamides **10**{*1*,*2*; *1*–*12*} were successfully obtained in 18–100% yields and in 80–100% purity. Out of 24 library members, 17 were ≥95% pure and 7 were ≥80% pure (Scheme 2, Table 1). 

**Table 1:** Selected experimental data for compounds **10**{*1*,*2*; *1–12*}.

Compd.	R^1^	R^2^R^3^NH 18	Workup [a]	Yield (%)	Purity (%)
**10**{*1*; *1*}	Me	1-pentylamine **18**{*1*}	B	85	80 [b]
**10**{*1*; *2*}	Me	cyclohexylamine **18**{*2*}	B	69	100 [b]
**10**{*1*; *3*}	Me	benzylamine **18**{*3*}	A	77	100 [b,c]
**10**{*1*; *4*}	Me	2-methoxyethylamine **18**{*4*}	A	28	100 [b,c]
**10**{*1*; *5*}	Me	3-amino-1-propanol **18**{*5*}	B	94	81 [b]
**10**{*1*; *6*}	Me	3-dimethylamino-1-propylamine **18**{*6*}	B	40	94 [b]
**10**{*1*; *7*}	Me	2-picolylamine **18**{*7*}	B	76	100 [b]
**10**{*1*; *8*}	Me	diethylamine **18**{*8*}	B	100	100 [b]
**10**{*1*; *9*}	Me	pyrrolidine **18**{*9*}	B	79	100 [b]
**10**{*1*; *10*}	Me	piperidine **18**{*10*}	B	100	100 [b]
**10**{*1*; *11*}	Me	morpholine **18**{*11*}	B	99	100 [b]
**10**{*1*; *12*}	Me	4-methylpiperazine **18**{*12*}	B	100	100 [b]
**10**{*2*; *1*}	Ph	1-pentylamine **18**{*1*}	A	100	100 [b,c]
**10**{*2*; *2*}	Ph	cyclohexylamine **18**{*2*}	A	100	86 [b,c]
**10**{*2*; *3*}	Ph	benzylamine **18**{*3*}	A	77	100 [b,c]
**10**{*2*; *4*}	Ph	2-methoxyethylamine **18**{*4*}	A	65	100 [b,c]
**10**{*2*; *5*}	Ph	3-amino-1-propanol **18**{*5*}	A	98	84 [b,c]
**10**{*2*; *6*}	Ph	3-dimethylamino-1-propylamine **18**{*6*}	A	71	100 [b]
**10**{*2*; *7*}	Ph	2-picolylamine **18**{*7*}	A	89	87 [b,c]
**10**{*2*; *8*}	Ph	diethylamine **18**{*8*}	B	68	88 [b,c]
**10**{*2*; *9*}	Ph	pyrrolidine **18**{*9*}	B	100	100 [b]
**10**{*2*; *10*}	Ph	piperidine **18**{*10*}	B	100	100 [b]
**10**{*2*; *11*}	Ph	morpholine **18**{*11*}	B	95	100 [b]
**10**{*2*; *12*}	Ph	4-methylpiperazine **18**{*12*}	B	100	100 [b]

[a] Workup A: filtration of the reaction mixture; Workup B: evaporation of the reaction mixture, followed by DFCC purification. [b] Determined by LC-MS, ^1^H-NMR, and ^13^C-NMR. [c] Confirmed by elemental analysis. The found values for C, H, and N were within ±0.4% range with respect to the theoretical values.

### 2.2. Structure Determination

The structures and purities of novel compounds **10**{*1*,*2*; *1–12*}, **16**{*1*,*2*}, and **17**{*1*,*2*} were determined by spectroscopic methods (IR, ^1^H-NMR and ^13^C-NMR, MS, HRMS), by LC-MS, and by elemental analyses for C, H, and N. Spectral and analytical data for novel compounds **10**{*1*,*2*; *1–12*}, **16**{*1*,*2*}, and **17**{*1*,*2*} were in agreement with the proposed structures. Correlation of NMR data for compounds **10**{*1*,*2*; *1–12*}, **16**{*1*,*2*}, and **17**{*1*,*2*} revealed very good agreement of chemical shifts and coupling constants for the core nuclei (Table 2).

Since the products **10**{*1*,*2*; *1*–*12*} were isolated as racemic mixtures [28], we also tried to find suitable conditions for separation of the enantiomers of compounds **10** by HPLC using analytical chiral stationary phase column Chiralcel^®^ OD-H (0.46 cm × 25 cm) and *n*-hexane/isopropanol as mobile phase. To our pleasant surprise, all 24 racemic compounds were resolved under these conditions. Most probably, the results obtained on analytical column should be applicable in (semi)preparative separation of enantiomers of **10**{*1*,*2*; *1−12*}, while these separation conditions could also serve as a important information for separation of analogous racemic compounds (Table 3).

Finally, some physicochemical properties of compounds **10**{*1*,*2*; *1–12*} were calculated to estimate their drug-likeness. The compounds have molecular weight (MW) between 160 and 500, number of atoms between 20 and 70, CLogP between –0.4 and 5.6, number of hydrogen bond donors (HBD) ≤ 5, number of hydrogen bond acceptors (HBA) ≤ 10, and polar surface area (PSA) bellow 140 Ǻ^2^ [29,30]. These calculated physicochemical properties compliant with Lipinski’s rule of five indicate promising drug-likeness of the synthesized compounds **10**{*1*,*2*; *1*–*12*} (Table 4). 

**Table 2:** Selected NMR data for compounds **10**{*1*,*2*; *1–12*}.

Compd.	δ (ppm)							^3^ *J*_H–H_ (Hz)					
	4-H	2'-Ha	2'-Hb	3'-H	4'-Ha	4'-Hb		2'a-2'b	2'a-3'	2'b-3'	3'-4'a	3'-4'b	4'a-4'b
**16**{*1*}	9.11	4.15	4.24	4.70	2.96	3.17		9.6	6.4	8.4	9.1	7.3	16.9
**16**{*2*}	9.28	4.16	4.36	4.78	3.04	3.29		9.7	5.4	8.1	8.9	6.2	16.9
**17**{*1*}	9.05	4.00	4.23	4.56	2.92 [a]			9.8	5.4	8.5	[a]	[a]	[a]
**17**{*2*}	9.25	4.06	4.35	4.73	2.96	3.03		9.9	4.1	7.9	4.9	8.6	16.7
**10**{*1; 1*}	8.62	4.14	4.23	4.28	2.90	3.15		9.3	6.5	7.3	8.8	7.3	17.0
**10**{*1; 2*}	8.60	4.16	4.22	4.27	2.91	3.15		9.1	6.5	8.7	8.8	7.5	16.9
**10**{*1; 3*}	8.63	4.09	4.18	4.28	2.83	3.10		9.5	6.8	8.9	9.0	7.7	16.9
**10**{*1; 4*}	8.65	4.15	4.21	4.28	2.90	3.16		9.4	7.1	8.6	8.9	7.9	16.8
**10**{*1; 5*}	8.65	4.16	4.22	4.31	2.92	3.12		9.6	6.8	8.9	9.0	7.7	16.9
**10**{*1; 6*}	8.61	4.14	4.25	4.43	2.92	3.18		9.6	6.7	8.4	9.1	7.8	16.9
**10**{*1; 7*}	8.78	4.16	4.22	4.34	2.93	3.19		9.5	7.1	8.9	9.0	8.0	16.9
**10**{*1; 8*}	8.49	4.13	4.20	3.84	2.88	[b]		8.5	8.5	8.5	8.8	[a]	16.7
**10**{*1; 9*}	8.56	4.17	4.21	3.96	2.90	3.17		9.5	8.4	7.6	9.0	8.7	16.9
**10**{*1;10*}	8.47	4.19 [a]		3.90	2.90	3.19		[a]	[a]	[a]	[a]	[a]	[a]
**10**{*1; 11*	8.48	4.19 [a]		3.91	2.90	3.18		[a]	[a]	[a]	[a]	[a]	[a]
**10**{*1;12*}	8.47	4.17 [a]		3.89	2.90	3.19		[a]	[a]	[a]	[a]	[a]	[a]
**10**{*2; 1*}	8.77	4.14	4.31	4.35	2.95	3.23		9.1	5.2	8.6	6.5	8.7	16.9
**10**{*2; 2*}	8.75	4.15	~4.3 [a]		2.95	3.23		9.6	4.8	[a]	8.8	6.5	16.9
**10**{*2; 3*}	8.81	4.14	4.31	4.39	2.96	3.26		9.6	5.8	8.8	8.8	6.7	16.9
**10**{*2; 4*}	8.82	4.17	4.32	4.37	2.98	3.27		9.4	5.8	8.8	8.6	6.8	16.8
**10**{*2; 5*}	8.80	4.17	4.31	4.38	2.98	3.21		9.6	5.6	8.9	8.7	6.5	16.9
**10**{*2; 6*}	8.77	4.20	4.28	4.51	3.00	3.29		9.7	5.8	8.1	8.9	6.7	16.9
**10**{*2; 7*}	8.96	4.19	4.32	4.44	3.00	3.29		9.6	6.1	8.3	8.9	7.1	16.9
**10**{*2; 8*}	8.65	4.22	4.24	3.92	2.95	[a]		[a]	[a]	[a]	8.9	[a]	16.9
**10**{*2; 9*}	8.72	4.24	4.27	4.06	2.97	3.28		9.7	6.8	8.2	8.9	7.8	16.9
**10**{*2;10*}	8.63	4.24	4.24	3.98	2.96	3.27		[a]	[a]	[a]	[a]	[a]	[a]
**10**{*2;11*}	8.64	4.23	4.25	3.99	2.97	3.27		[a]	[a]	[a]	8.3	7.0	16.3
**10**{*2;12*}	8.64	4.25	4.25	3.98	2.98	3.29		[a]	[a]	[a]	[a]	[a]	[a]

[a] Multiplet or broad singlet; [b] Overlapped by other signals.

**Table 3:** Analytical data for separation of enantiomers of racemic compounds **10**{*1*,*2*; *1–12*}.

Compound	*n*-hexane:*i*-PrOH	R *t* (min)		
		Enantiomer A		Enantiomer B
**10**{*1*; *1*}	50:50	4.084		5.078
**10**{*1*; *2*}	50:50	9.987		16.650
**10**{*1*; *3*}	50:50	8.208		12.734
**10**{*1*; *4*}	50:50	5.321		7.031
**10**{*1*; *5*}	50:50	3.828		4.542
**10**{*1*; *6*}	50:50	5.083		5.477
**10**{*1*; *7*}	50:50	7.577		8.352
**10**{*1*; *8*}	50:50	5.728		6.380
**10**{*1*; *9*}	50:50	6.960		9.471
**10**{*1*; *10*}	50:50	5.798		7.185
**10**{*1*; *11*}	50:50	8.509		9.619
**10**{*1*; *12*}	50:50	7.206		7.928
**10**{*2*; *1*}	50:50	4.409		5.515
**10**{*2*; *2*}	50:50	4.537		5.840
**10**{*2*; *3*}	50:50	11.292		29.227
**10**{*2*; *4*}	50:50	6.462		7.522
**10**{*2*; *5*}	80:20	14.864		18.975
**10**{*2*; *6*}	50:50	6.160		19.660
**10**{*2*; *7*}	50:50	10.778		12.764
**10**{*2*; *8*}	50:50	5.293		24.904
**10**{*2*; *9*}	50:50	9.284		10.960
**10**{*2*; *10*}	50:50	7.102		8.212
**10**{*2*; *11*}	80:20	14.864		18.975
**10**{*2*; *12*}	50:50	14.429		19.625

**Table 4:** Calculated physicochemical properties of compounds 10{*1,2; 1–12*} [a].

Compound	MW (g·mol^–1^)	No. of atoms	CLogP	No. of HBD	No. of HBA	PSA (Ǻ^2^)
**10**{*1*; *1*}	366	53	2.82	1	6	74
**10**{*1*; *2*}	378	54	2.73	1	6	74
**10**{*1*; *3*}	386	51	2.67	1	6	74
**10**{*1*; *4*}	354	48	0.90	1	7	83
**10**{*1*; *5*}	354	48	0.46	2	7	94
**10**{*1*; *6*}	381	55	1.39	1	7	77
**10**{*1*; *7*}	387	50	1.17	1	7	86
**10**{*1*; *8*}	352	50	1.63	0	6	65
**10**{*1*; *9*}	350	48	1.20	0	6	65
**10**{*1*; *10*}	364	51	1.76	0	6	65
**10**{*1*; *11*}	366	49	0.73	0	7	75
**10**{*1*; *12*}	379	53	1.29	0	7	69
**10**{*2*; *1*}	428	60	4.42	1	6	74
**10**{*2*; *2*}	440	61	4.33	1	6	74
**10**{*2*; *3*}	448	58	4.27	1	6	74
**10**{*2*; *4*}	416	55	2.50	1	7	83
**10**{*2*; *5*}	416	55	2.06	2	7	94
**10**{*2*; *6*}	443	62	2.99	1	7	77
**10**{*2*; *7*}	449	57	2.77	1	7	86
**10**{*2*; *8*}	414	57	3.22	0	6	65
**10**{*2*; *9*}	412	55	2.80	0	6	65
**10**{*2*; *10*}	426	58	3.36	0	6	65
**10**{*2*; *11*}	428	56	2.33	0	7	75
**10**{*2*; *12*}	441	60	2.89	0	7	69

[a] Calculated with ChemBioDraw Ultra v11.0.

## 3. Experimental

### 3.1. General Methods

Melting points were determined on a Stanford Research Systems MPA100 OptiMelt automated melting point system (Sunnyvale, CA, USA). The NMR spectra were obtained on a Bruker Avance III UltraShield 500 plus (Karlsruhe, Germany) at 500 MHz for ^1^H and 126 MHz for the ^13^C nucleus, using DMSO-d_6_ and CDCl_3_ with TMS as the internal standard, as solvents. Mass spectra were recorded on a Agilent 6224 Accurate Mass TOF LC/MS spectrometer (Santa Clara, CA, USA), IR spectra on a Perkin-Elmer Spectrum BX FTIR spectrophotometer (Waltham, MA, USA). Microanalyses were performed on a Perkin-Elmer CHN Analyzer 2400 II (Waltham, MA, USA). Drying of the compounds **10** and **17** was performed in a Büchi drying oven (Flawil, Switzerland). Dry flash column chromatography (DFCC) was performed on Aluminium Oxide Fluka for Chromatography, cat. # 06310, type 506 C weakly acidic, 0.05–0.15 mm, pH 6.0 ± 0.5 (Buchs, Switzerland).

For LC-MS/MS experiments, liquid chromatograph Perkin Elmer Series 200 from Perkin Elmer (Shelton, CT, USA) with UV detector and 3200 QTRAP LC/MS/MS System equipped with ESI and APCI ion sources from Applied Biosystems/MDS Sciex (Foster City, CA, USA) were used. HPLC column was Gemini, dimensions 150 mm × 4.6 mm, 3 μm particles from Phenomenex (Torrance, CA, USA). Mobile phase was a gradient of acetonitrile (A) and deionised water (B): 0 min-10% A, 25 min-100% A, 3 min equilibration time with initial mobile phase (10% A) was allowed for column equilibration. Mobile phase flow was 1 mL/min. Injection volume was 20 μL. Signal was recorded using UV detector at 254 nm and mass spectra were recorded using positive (ESI+) and negative (ESI−) ionization mode simultaneously. Mass range was from 70 to 500 amu. Electrospray ion source (ESI) conditions were as follows: cone voltage 5500 V (ESI+) and −4500 V (ESI−), respectively, ion source temperature 4,000 °C, curtain gas N_2_ pressure was set to 10 psi, nebulizer gas N_2_ pressure was set to 20 psi and turbo gas (air) pressure was set to 40 psi. Declustering potential 30 V and entrance potential 10 V was used, respectively.

Itaconic acid (**11**), 1,1'-carbonyldiimidazole, *N*,*N*-dimethylformamide dimethylacetal (DMFDMA), acetamidine hydrochloride **15**{*1*}, benzamidine **15**{*2*}, bis(pentafluorophenyl) carbonate (BPC), and amines **18**{*1*–*12*} are commercially available (Sigma-Aldrich). 5-Oxo-1-phenylpyrrolidin-3-carboxylic acid (**12**) [24] and methyl 3-oxo-3-(5-oxo-1-phenylpyrrolidin-3-yl)propanoate (**13**) [23] were prepared according to the literature procedures.

Parallel stirring and filtrations were carried out on Mettler-Toledo Bohdan MiniBlock™ Compact Shaking and Washing Station and Vacuum Collection Base (2 × 12 positions, Vortex stirring, 400 r.p.m. in all cases). Parallel evaporations were carried out on Büchi Syncore® Polyvap parallel evaporator (24 positions, Vortex stirring, 400 r.p.m. in all cases). Parallel drying was carried out on Hettlab IR-Dancer Infra-Red Vortex-Evaporator (42 positions, Vortex stirring, 400 r.p.m. in all cases).

### 3.2. Synthesis of Methyl 3-(Dimethylamino)-2-(5-oxo-1-phenylpyrrolidine-3-carbonyl)acrylate *(**14**)*

This compound was prepared according to a slightly modified literature procedure [23]. A mixture of *β*-keto ester **13** [23] (5.2 g, 20 mmol), anhydrous toluene (20 mL), and DMFDMA (2.8 g, 3 mL, 20 mmol) was stirred at 60 °C for 3 h. Volatile components were evaporated *in vacuo* to give the crude **14** as a yellow oil in quantitative yield.

### 3.3. Synthesis of Methyl 2-Methyl-6-(5-Oxo-1-phenylpyrrolidin-3-yl)pyrimidine-5-carboxylate *(**16**{*1*})*

Cold (0 °C) solution of *t*-BuOK (2.3 g, 20 mmol) in anhydrous methanol (20 mL) was added to a cold (0 °C) solution of acetamidine hydrochloride (**15**{*1*}, 1.9 g, 20 mmol) in methanol (20 mL) and the mixture was stirred at 0 °C for 5 min. The suspension was filtered with suction through a fritted funnel and the precipitated KCl was washed with anhydrous methanol (2 × 10 mL) to afford a solution of the free acetamidine **15**{*1*} (20 mmol) in methanol. This was added to a solution of the crude enaminone **13** (20 mmol) in methanol (100 mL) and the mixture was stirred at room temperature for 18 h. The precipitate was collected by filtration, and washed with methanol (2 × 10 mL) to give **16**{*1*}. Yield: 3.2 g (50%) of white solid; m.p. 131–133 °C. ^1^H-NMR (500 MHz, DMSO-d_6_): δ 2.77 (3H, s, 2-CH_3_); 2.96 (1H, dd, *J* = 9.1, 16.9 Hz, 4'-Ha); 3.17 (1H, dd, *J* = 7.3, 16.9 Hz, 4'-Hb); 3.97 (3H, s, OCH_3_); 4.15 (1H, dd, *J* = 6.4, 9.6 Hz, 2'-Ha); 4.24 (1H, dd, *J* = 8.4, 9.5 Hz, 2'-Hb); 4.70 (1H, quintet, *J* = 8.3 Hz, 3'-H); 7.14 (1H, br t, *J* = 7.4 Hz, *p*-Ph); 7.37 (2H, br t, *J* = 8.0 Hz, *o*-Ph); 7.63 (2H, br d, *J* = 7.9 Hz, *m*-Ph); 9.06 (1H, s, 4-H). ^13^C-NMR (126 MHz, CDCl_3_): δ 26.5, 35.5, 38.0, 52.9, 53.3, 120.1, 120.3, 124.8, 129.0, 139.3, 159.5, 165.2, 170.1, 171.2, 172.7. LC-MS: R*_t_* = 13.217 min, *m*/*z* = 312 (MH^+^), area% = 80. *m*/*z* (HRMS) Found: 312.1345 (MH^+^). C_17_H_18_N_3_O_3_ requires: *m*/*z* = 312.1343. (Found: C, 65.22; H, 5.46; N, 13.16. C_17_H_17_N_3_O_3_ requires: C, 65.58; H, 5.50; N, 13.50.); *ν*_max_ (KBr) 3420, 1718, 1693, 1598, 1572, 1545, 1480, 1397, 1306, 1268, 1097, 818, 764, 693 cm^−1^. 

### 3.4. Synthesis of Methyl 6-(5-Oxo-1-phenylpyrrolidin-3-yl)-2-phenylpyrimidine-5-carboxylate *(**16**{*2*})*

Benzamidine **15**{*2*} (2.4 g, 20 mmol) was added to a solution of the crude enaminone **14** (20 mmol) in anhydrous methanol (100 mL) and the mixture was stirred at room temperature for 72 h. The precipitate was collected by filtration, and washed with methanol (2 × 30 mL) to give **16**{*2*}. Yield: 4.9 g (65%) of white solid; m.p. 146–147 °C. ^1^H-NMR (500 MHz, CDCl_3_): δ 3.04 (1H, dd, *J* = 8.9, 16.9 Hz, 4'-Ha); 3.29 (1H, dd, *J* = 6.2, 16.9 Hz, 4'-Hb); 4.00 (3H, s, OCH_3_); 4.16 (1H, dd, *J* = 5.4, 9.7 Hz, 2'-Ha); 4.36 (1H, dd, *J* = 8.1, 9.6 Hz, 2'-Hb); 4.78 (1H, tt, *J* = 5.9, 8.4 Hz, 3'-H); 7.16 (1H, t, *J* = 7.4 Hz, *p-*Ph); 7.38 (2H, br t, *J* = 8.0 Hz, *o-*Ph); 7.47–7.55 (3H, m, 3H of Ph); 7.64 (2H, br d, *J* = 7.7 Hz, *m-*Ph); 8.50–8.52 (2H, m, *m*-Ph); 9.28 (1H, s, 4-H). ^13^C-NMR (126 MHz, CDCl_3_): δ 35.9, 38.0, 52.9, 53.6, 120.2, 120.3, 124.8, 128.9, 129.0, 129.2, 132.2, 136.3, 139.3, 160.1, 165.1, 166.2, 170.5, 173.0. LC-MS: R*_t_* = 19.692 min, *m*/*z* = 374 (MH^+^), area% = 100. *m*/*z* (ESI) = 374 (MH^+^). *m*/*z* (HRMS) Found: 374.1499 (MH^+^). C_22_H_20_N_3_O_3_ requires: *m*/*z* = 374.1499. (Found: C, 69.61; H, 5.44; N, 11.07. C_22_H_19_N_3_O_3_·⅓H_2_O requires: C 69.59; H 5.23; N 11.07.); *ν*_max_ (KBr) 3484, 1717, 1676, 1569, 1478, 1406, 1311, 1281, 1196, 1108, 836, 766, 692 cm^−1^.

### 3.5. Synthesis of 2-Methyl-6-(5-oxo-1-phenylpyrrolidin-3-yl)pyrimidine-5-carboxylic Acid *(**17**{*1*})*

A mixture of the ester **16** (13 mmol), 1 M aqueous NaOH (30 mL), methanol (30 mL), and THF (30 mL) was stirred at room temperature for 5 h. Methanol and THF were removed by evaporation *in vacuo* (35 °C, 100 mbar), the aqueous residue was acidified with concentrated hydrochloric acid to pH ~1, and the product was extracted with dichloromethane (4 × 200 mL). The combined organic phases were dried over anhydrous Na_2_SO_4_, filtered, and the filtrate was evaporated in vacuo to give **17**{*1*}. Yield: 3.33 g (86%) of a pale yellow solid; m.p. 70–82 °C. ^1^H-NMR (500 MHz, DMSO-d_6_): δ 2.66 (3H, s, 2-CH_3_); 2.88–2.96 (2H, m, 4'-CH_2_); 4.00 (1H, dd, *J* = 5.4, 9.8 Hz, 2'-Ha); 4.23 (1H, dd, *J* = 8.5, 9.7 Hz, 2'-Hb); 4.56 (1H, dq, *J* = 5.6, 8.0 Hz, 3'-H); 7.13 (1H, br t, *J* = 7.4 Hz, *p-*Ph); 7.37 (2H, br t, *J* = 8.0 Hz, *o-*Ph); 7.65 (2H, br d, *J* = 7.8 Hz, *m-*Ph); 9.05 (1H, s, 4-H); 13.78 (1H, s, COOH). ^13^C (126 MHz, DMSO-d_6_): δ 26.0, 34.4, 37.4, 52.6, 119.6, 121.0, 124.0, 128.7, 139.3, 159.1, 166.0, 169.6, 170.0, 172.3. LC-MS: *R*t = 13.7 min, *m*/*z* = 296 (M–H^+^), area% = 100. *m*/*z* (ESI) = 296 (M–H^+^). *m*/*z* (HRMS) Found: 298.1189 (MH^+^). C_16_H_16_N_3_O_3_ requires: *m*/*z* = 298.1186. (Found: C 64.51; H 5.16; N 13.95. C_16_H_15_N_3_O_3_ requires: C 64.64; H 5.09; N 14.13.); *ν*_max_ (KBr) 3418, 1700, 1676, 1597, 1542, 1500, 1400, 1265, 762, 692 cm^−1^.

### 3.6. Synthesis of 6-(5-Oxo-1-phenylpyrrolidin-3-yl)-2-phenylpyrimidine-5-carboxylic Acid *(**17**{*2*})*

A mixture of the ester **16** (13 mmol), 1 M aqueous NaOH (30 mL), methanol (30 mL), and THF (30 mL) was stirred at room temperature for 5 h. Methanol and THF were removed by evaporation *in vacuo* (35 °C, 100 mbar) and the aqueous residue was acidified with concentrated hydrochloric acid to pH ~1. The precipitate was collected by filtration to give **17**{*2*}. Yield: 4.34 g (92%) of a pale yellow solid; m.p. 251–253 °C. ^1^H-NMR (500 MHz, DMSO-d_6_): δ 2.96 (1H, dd, *J* = 4.9, 16.7 Hz, 4'-Ha); 3.03 (1H, dd, *J* = 8.6, 16.7 Hz, 4'-Hb); 4.06 (1H, dd, *J* = 4.1, 9.9 Hz, 2'-Ha); 4.35 (1H, dd, *J* = 7.9, 9.9 Hz, 2'-Hb); 4.73 (1H, br septet, *J* = 4.2 Hz, 3'-H); 7.13 (1H, br t, *J* = 7.4 Hz, *p-*Ph); 7.37 (2H, br t, *J* = 8.0 Hz, *o-*Ph); 7.52 (2H, br t, *J* = 8.0 Hz, *o-*Ph); 7.57 (1H, br t, *J* = 7.3 Hz, *p-*Ph); 7.69 (2H, br d, *J* = 7.8 Hz, *m-*Ph); 8.43 (2H, br d, *J* = 7.2 Hz, *m-*Ph); 9.25 (1H, s, 4-H); 13.84 (1H, s, COOH). ^13^C (126 MHz, DMSO-d_6_): δ 35.0, 37.7, 52.8, 119.5, 121.2, 123.9, 128.4, 128.7, 128.8, 131.9, 136.2, 139.5, 159.8, 164.3, 165.9, 170.8, 172.6. LC-MS: *R*t = 18.192 min, *m*/*z* = 358 (M–H^+^), area% = 100. *m*/*z* (ESI) = 358 (M–H^+^). *m*/*z* (HRMS) Found: 360.1346 (MH^+^). C_21_H_18_N_3_O_3_ requires: *m*/*z* = 360.1343. (Found: C 69.38; H 4.65; N 11.38. C_21_H_17_N_3_O_3_·¼H_2_O requires: C 69.37; H 4.84; N 11.56.); *ν*_max_ (KBr) 3420, 2364, 1708, 1654, 1567, 1500, 1424, 1312, 1257, 1183, 756, 697 cm^−1^.

### 3.7. Parallel Synthesis of 2-Substituted 6-(5-Oxo-1-phenylpyrrolidin-3-yl)pyrimidine-5-carboxamides ***10**{*1*,*2*;* 1–12*}*

Two MiniBlocks^TM^ were equipped with 12 fritted vessels each and mounted on a compact stirring and washing station. The reaction vessels were charged with carboxylic acids **17**{*1*} (12 × 149 mg, 12 × 0.5 mmol) and **17**{*2*} (12 × 180 mg, 12 × 0.5 mmol), anhydrous acetonitrile (24 × 5 mL), BPC (24 × 236 mg, 24 × 0.6 mmol), and triethylamine (24 × 0.14 mL, 24 × 1 mmol) and the mixtures were stirred at room temperature for 30 min. Then, the amines **18**{*1–7*} (2 × 12 × 0.5 mmol) and amines **18**{*8–12*} (2 × 4 × 5 mmol) were added and stirring at room temperature was continued for 12 h. The reaction mixtures were filtered to afford **10**{*1*; *3*,*4*} and **10**{*2*; *1*–*7*} (Workup A). The filtrates containing the products **10**{*1*; *1*,*2*,*5–12*} and **10**{*2*; *8–12*} were evaporated *in vacuo* (40 °C/2 mbar) and the residues (resins) were dissolved in dichloromethane (15 × 2.5 mL) and purified sequentially by DFCC over aluminium oxide (5 g, d = 15 mm) by gradient elution with a) EtOAc (30 mL) and b) EtOAc–EtOH (5:1, 50 mL). The combined eluates were evaporated *in vacuo* (60 °C/1 mbar) to afford compounds **10**{*1*; *1*,*2*,*5–12*} and **10**{*2*; *8*–*12*} (Workup B). The following compounds were prepared in this manner.

#### 3.7.1. *2-Methyl-6-(5-oxo-1-phenylpyrrolidin-3-yl)-N-pentylpyrimidine-5-carboxamide* (**10**{*1*; *1*})

Prepared from **17**{*1*} and 1-pentylamine (**18**{*1*}), workup B. Yield: 204 mg (85%) of yellow-brown resin. ^1^H-NMR (500 MHz, CDCl_3_): δ 0.92 (3H, dd, *J* = 4.7, 9.0 Hz, CH_3_ of C_5_H_11_), 1.35–1.40 (4H, m, 2CH_2_ of C_5_H_11_), 1.64 (2H, quintet, *J* = 7.3 Hz, CH_2_ of C_5_H_11_), 2.74 (3H, s, 2-CH_3_), 2.90 (1H, dd, *J* = 8.8, 17.0 Hz, 4'-Ha), 3.15 (1H, dd, *J* = 7.3, 17.0 Hz, 4'-Hb), 3.45 (2H, br q, *J* = 7.3 Hz, C*H*_2_NH), 4.14 (1H, dd, *J* = 6.5, 9.3 Hz, 2'-Ha), 4.23 (1H, t, *J* = 8.8 Hz, 2'-Hb), 4.28 (1H, br q, *J* = 8.2 Hz, 3'-H), 6.24 (1H, s, NH), 7.15 (1H, br t, *J* = 7.4 Hz, *p-*Ph), 7.36 (2H, br t, *J* = 7.9 Hz, *m-*Ph), 7.59 (2H, br d, *J* = 7.6 Hz, *o*-Ph), 8.62 (1H, s, 4-H). ^13^C-NMR (126 MHz, CDCl_3_): δ 14.2, 22.5, 26.3, 29.3, 29.4, 35.7, 38.3, 40.6, 53.9, 120.6, 125.1, 126.5, 129.1, 139.1, 154.7, 165.9, 168.3, 169.8, 172.9. LC-MS: *R*t = 14.733 min, *m*/*z* = 367 (MH^+^), area% = 80. *m*/*z* (ESI) = 365 (M−H^+^). *m*/*z* (HRMS) Found: 365.1987 ([M−H]^−^). C_21_H_25_N_4_O_2_ requires: *m*/*z* = 365.1983. *ν*_max_ (KBr) 3460, 2356, 1651, 1516, 1501, 985, 760, 694 cm^−1^.

#### 3.7.2. *N-Cyclohexyl-2-methyl-6-(5-oxo-1-phenylpyrrolidin-3-yl)pyrimidine-5-carboxamide* (**10**{*1*; *2*})

Prepared from **17**{*1*} and (cyclohexylamine **18**{*2*}), workup B. Yield: 228 mg (69%) of yellow-brown resin. ^1^H-NMR (500 MHz, CDCl_3_): δ 1.16–1.32 (4H, m, 4H of C_6_H_11_), 1.43 (2H, br tq, *J* = 3.5, 12.1 Hz, 2H of C_6_H_11_), 1.68 (1H, br td, *J* = 3.5, 12.8 Hz, 2H of C_6_H_11_), 1.75–1.81 (2H, m, 2H of C_6_H_11_), 2.05 (2H, br dd, *J* = 2.2, 12.3 Hz, 2H of C_6_H_11_), 2.74 (3H, s, 2-CH_3_), 2.91 (1H, dd, *J* = 8.8, 16.9 Hz, 4'-Ha ), 3.15 (1H, dd, *J* = 7.5, 16.9 Hz, 4'-Hb), 3.95 (1H, ttd, *J* = 3.9, 7.8, 14.5 Hz, 1H of C_6_H_11_), 4.16 (1H, dd, *J* = 6.5, 9.1 Hz, 2'-Ha), 4.22 (1H, t, *J* = 8.7 Hz, 2'-Hb), 4.27 (1H, quintet, *J* = 8.0 Hz, 3'-H), 6.04 (1H, *J* = 6.8 Hz, NH), 7.16 (1H, br t, *J* = 7.8 Hz, *p*-Ph), 7.36 (2H, br t, *J* = 8.0 Hz, *m*-Ph), 7.59 (2H, br d, *J* = 7.7 Hz, *o*-Ph), 8.60 (1H, s, 4-H). ^13^C-NMR (126 MHz, CDCl_3_): δ 25.0, 25.6, 26.2, 33.2, 35.7, 38.3, 49.6, 53.9, 120.6, 125.1, 126.7, 129.1, 139.1, 154.6, 165.1, 168.2, 169.7, 173.0. LC-MS: *R*t = 14.483 min, *m*/*z* = 379 (MH^+^), area% = 100. *m*/*z* (ESI) = 379 (MH^+^). *m*/*z* (HRMS) Found: 379.2131 (MH^+^). C_22_H_27_N_4_O_2_ requires: *m*/*z* = 379.2129. *ν*_max_ (KBr) 3418, 2934, 1634, 1516, 1501, 1400, 1281, 1150, 1007, 839, 762, 694 cm^−1^.

#### 3.7.3. *N-Benzyl-2-methyl-6-(5-oxo-1-phenylpyrrolidin-3-yl)pyrimidine-5-carboxamide* (**10**{*1*; *3*})

Prepared from **17**{*1*} and benzylamine (**18**{*3*}), workup A. Yield: 150 mg (77%) of white solid; m.p. 153–155 °C. ^1^H-NMR (500 MHz, CDCl_3_): δ 2.72 (3H, s, 2-CH_3_), 2.83 (1H, dd, *J* = 9.0, 16.9 Hz, 1H, 4'-Ha), 3.10 (1H, dd, *J* = 7.7, 16.9 Hz, 4'-Hb), 4.09 (1H, dd, *J* = 6.8, 9.5 Hz, 2'-Ha), 4.18 (1H, t, *J* = 8.9 Hz, 2'-Hb), 4.28 (1H, quintet, *J* = 7.9 Hz, 3'-H), 4.59 and 4.63 (2H, 2dd, 1:1, *J* = 5.5, 14.5 Hz, C*H*_2_Ph), 6.72 (1H, t, *J* = 5.3 Hz, NH), 7.14 (1H, br t, *J* = 7.4 Hz, *p*-Ph), 7.31–7.38 (7H, m, *m-*Ph, Ph'), 7.55 (2H, br d, *J* = 7.4 Hz, *o*-Ph), 8.63 (1H, s, 4-H). ^13^C-NMR (126 MHz, CDCl_3_): δ 26.4, 35.6, 38.3, 44.6, 53.9, 120.5, 125.0, 126.2, 128.2, 128.3, 129.1, 129.2, 137.6, 139.2, 155.0, 165.9, 168.3, 170.0, 172.9. LC-MS: *R*t = 13.808 min, *m*/*z* = 387 (MH^+^), area% = 100. *m*/*z* (ESI) = 385 (M–H^+^). *m*/*z* (HRMS) Found: 385.1669 ([M−H]^−^). C_23_H_21_N_4_O_2_ requires: *m*/*z* = 385.167. (Found: C 71.30; H 5.83; N 14.44. C_23_H_22_N_4_O_2_ requires: C 71.48; H 5.74; N 14.50); *ν*_max_ (KBr) 3422, 3269, 1706, 1634, 1560, 1498, 1446, 1396, 1308, 1279, 1218, 824, 756, 691 cm^−1^.

#### 3.7.4. *N-(2-Methoxyethyl)-2-methyl-6-(5-oxo-1-phenylpyrrolidin-3-yl)pyrimidine-5-carboxamide* (**10**{*1*; *4*})

Prepared from **17**{*1*} and 2-methoxyethylamine (**18**{*4*}), workup A. Yield: 48 mg (28%) of white solid; m.p. 101–103 °C. ^1^H-NMR (500 MHz, CDCl_3_): δ 2.74 (3H, s, 2-CH_3_), 2.90 (1H, dd, *J* = 8.9, 16.8 Hz, 1H, 4'-Ha), 3.16 (1H, dd, *J* = 7.9, 16.8 Hz, 4'-Hb), 3.39 (3H, s, OCH_3_), 3.57–3.59 and 3.63–3.66 (4H, 2m, 1:1, CH_2_CH_2_), 4.15 (1H, dd, *J* = 7.1, 9.4 Hz, 2'-Ha), 4.21 (1H, t, *J* = 8.6 Hz, 2'-Hb), 4.28 (1H, quintet, *J* = 8.1 Hz, 3'-H), 6.25 (1H, s, NH), 7.15 (1H, br t, *J* = 7.4 Hz, *p*-Ph), 7.36 (2H, br t, *J* = 8.0 Hz, *m*-Ph), 7.61 (2H, br d, *J =* 8.5 Hz, *o*-Ph), 8.65 (1H, s, 4-H). ^13^C-NMR (126 MHz, CDCl_3_): δ 26.3, 35.7, 38.3, 40.1, 53.8, 59.1, 70.9, 120.4, 120.4, 124.9, 126.2, 129.0, 129.0, 139.2, 155.0, 166.0, 168.1, 169.9, 172.8. LC-MS: *R*t = 10.167 min, *m*/*z* = 355 (MH^+^), area% = 100. *m*/*z* (ESI) = 355 (MH^+^). *m*/*z* (HRMS) Found: 389.1387 ([M+Cl]^−^). C_19_H_22_ClN_4_O_3_ requires: *m*/*z* = 389.1386. (Found: C 64.17; H 6.22; N 15.67. C_19_H_22_N_4_O_3_ requires: C 64.39; H 6.26; N 15.81); *ν*_max_ (KBr) 3422, 3269, 1706, 1634, 1560, 1498, 1446, 1396, 1308, 1279, 1218, 824, 756, 691 cm^−1^.

#### 3.7.5. *N-(3-Hydroxypropyl)-2-methyl-6-(5-oxo-1-phenylpyrrolidin-3-yl)pyrimidine-5-carboxamide* (**10**{*1*; *5*})

Prepared from **17**{*1*} and 3-amino-1-propanol (**18**{*5*}), workup B. Yield: 205 mg (94%) of yellow-brown resin. ^1^H-NMR (500 MHz, CDCl_3_): δ 1.85 (2H, quintet, *J* = 6.3 Hz, CH_2_C*H*_2_CH_2_), 2.73 (3H, s, 2-CH_3_), 2.92 (1H, dd, *J* = 9.0, 16.9 Hz, 4'-Ha), 3.12 (1H, dd, *J* = 7.7, 16.9 Hz, 4'-Hb), 3.62 (2H, ddd, *J* = 2.2, 5.6, 11.5 Hz, C*H*_2_NH), 3.81 (2H, t, *J* = 5.5 Hz, C*H*_2_OH), 4.16 (1H, dd, *J* = 6.8, 9.6 Hz, 2'-Ha), 4.22 (1H, t, *J* = 8.9 Hz, 2'-Hb), 4.31 (1H, quintet, *J* = 7.9 Hz, 3'-H), 6.40 (1H, br s, NH), 7.16 (1H, br t, *J* = 7.5 Hz, *p-*Ph), 7.19 (1H, br t, *J* = 5.0 Hz, OH), 7.36 (2H, br t, *J* = 8.0 Hz, *m*-Ph), 7.58 (2H, d, *J* = 7.8 Hz, *o*-Ph), 8.65 (1H, s, 4-H). ^13^C-NMR (126 MHz, CDCl_3_): δ 26.2, 31.4, 35.6, 38.4, 38,7, 54.0, 61.2, 120.7, 125.2, 126.3, 129.1, 139.0, 155.0, 166.4, 168.3, 169.8, 173.1. LC-MS: *R*t = 9.183 min, *m*/*z* = 355 (MH^+^), area% = 81. *m*/*z* (ESI) = 353 ([M−H]^−^). *m*/*z* (HRMS) Found: 353.1623 ([M−H]^−^). C_19_H_21_N_4_O_3_ requires: *m*/*z* = 353.1619. *ν*_max_ (KBr) 3444, 2370, 1645, 1517, 1501, 1309, 984, 838, 761, 691 cm^−1^.

#### 3.7.6. *N-(3-Dimethylaminopropyl)-2-methyl-6-(5-oxo-1-phenylpyrrolidin-3-yl)pyrimidine-5-carboxamide* (**10**{*1*; *6*})

Prepared from **17**{*1*} and 3-(dimethylamino)propylamine (**18**{*6*}), workup B. Yield: 115 mg (40%) of yellow resin. ^1^H-NMR (500 MHz, CDCl_3_): δ 1.79 (2H, br quintet, *J* = 5.8 Hz, CH_2_C*H*_2_CH_2_), 2.28 (6H, s, NMe_2_), 2.54 (2H, t, *J* = 5.7 Hz, C*H*_2_NMe_2_), 2.74 (3H, s, 2-CH_3_), 2.92 (1H, dd, *J* = 9.1, 16.9 Hz, 4'-Ha), 3.18 (1H, dd, *J* = 7.8, 16.9 Hz, 4'-Hb), 3.56 (2H, tq, *J* = 6.0, 7.0 Hz, C*H*_2_NH), 4.14 (1H, dd, *J* = 6.7, 9.6 Hz, 2'-Ha), 4.25 (1H, dd, *J* = 8.4, 9.6 Hz, 2'-Hb), 4.43 (1H, quintet, *J* = 8.2 Hz, 3'-H), 7.15 (1H, br t, *J* = 7.4 Hz, *p*-Ph), 7.36 (2H, br t, *J* = 8.0 Hz, *m*-Ph), 7.62 (2H, br d, *J* = 7.7 Hz, *o*-Ph), 8.61 (1H, s, NH), 8.78 (1H, br s, 4-H). ^13^C-NMR (126 MHz, CDCl_3_): δ 24.6, 26.2, 35.8, 37.6, 38.5, 43.7, 53.9, 56.0, 120.5, 125.0, 126.0, 129.0, 139.2, 155.3, 166.5, 168.2, 169.7, 173.0. LC-MS: *R*t = 1.867 min, *m*/*z* = 382 (MH^+^), area% = 94. *m*/*z* (ESI) = 353 (MH^+^). *m*/*z* (HRMS) Found: 382.2242 (MH^+^). C_21_H_28_N_5_O_2_ requires: *m*/*z* = 382.2238. *ν*_max_ (KBr) 3444, 2356, 1651, 1503, 1312, 1163, 1008, 985, 838, 762, 693 cm^−1^.

#### 3.7.7. *2-Methyl-6-(5-oxo-1-phenylpyrrolidin-3-yl)-N-((pyridin-2-yl)methyl)pyrimidine-5-carboxamide* (**10**{*1*; *7*})

Prepared from **17**{*1*} and 2-picolylamine (**18**{*7*}), workup B. Yield: 214 mg (76%) of yellow-brown resin. ^1^H-NMR (500 MHz, CDCl_3_): δ 2.76 (3H, s, 2-CH_3_), 2.93 (1H, dd, *J* = 9.0, 16.9 Hz, 4'-Ha), 3.19 (1H, dd, *J* = 8.0, 16.9 Hz, 4'-Hb), 4.16 (1H, dd, *J* = 7.1, 9.5 Hz, 2'-Ha), 4.22 (1H, t, *J* = 8.9 Hz, 2'-Hb), 4.34 (1H, quintet, *J* = 8.9 Hz, 3'-H), 4.74 and 4.78 (2H, 2dd, 1:1, *J* = 4.8, 16.5 Hz, C*H*_2_NH), 7.15 (1H, br t, *J* = 7.4 Hz, *p-*Ph), 7.28 (1H, dd, *J* = 5.3, 7.2 Hz, 5''-H), 7.36 (3H, br t, *J* = 8.0 Hz, *m*-Ph, NH), 7.58–7.64 (3H, m, *o*-Ph, 3''-H), 7.75 (1H, dt, *J* = 1.7, 7.7 Hz, 4''-H), 8.53 (1H, br d, *J* = 4.6 Hz, 6''-H), 8.78 (1H, s, 4-H). ^13^C-NMR (126 MHz, CDCl_3_): δ 26.3, 35.7, 38.4, 44.7, 53.9, 120.5, 122.8, 123.2, 125.0, 126.3, 129.1, 137.7, 139.2, 149.1, 155.0, 155.2, 165.9, 168.3, 170.0, 173.0. LC-MS: *R*t = 11.775 min, *m*/*z* = 388 (MH^+^), area% = 100. *m*/*z* (ESI) = 388 (MH^+^). *m*/*z* (HRMS) Found: 388.1769 (MH^+^). C_22_H_22_N_5_O_2_ requires: *m*/*z* = 388.1768. *ν*_max_ (KBr) 3452, 1654, 1515, 1500, 1405, 1311, 986, 760, 696 cm^−1^.

#### 3.7.8. *N,N-(Diethyl)-2-methyl-6-(5-oxo-1-phenylpyrrolidin-3-yl)pyrimidine-5-carboxamide* (**10**{*1*; *8*})

Prepared from **17**{*1*} and diethylamine (**18**{*8*}), workup B. Yield: 219 mg (100%) of yellow resin. ^1^H-NMR (500 MHz, CDCl_3_): δ 1.14 and 1.29 (6H, 2t, 1:1, *J* = 7.1 Hz, 2CH_2_C*H*_3_), 2.75 (3H, s, 2-CH_3_), 2.86 (1H, dd, *J* = 8.8, 16.7 Hz, 4'-Ha), 3.15–3.27 (3H, m, 4'-Hb, C*H*_2_CH_3_), 3.53–3.67 (2H, m, C*H*_2_CH_3_), 3.83 (1H, quintet, *J* = 8.6 Hz, 3'-H), 4.12 (1H, br t, *J* = 8.5 Hz, 2'-Ha), 4.20 (1H, br t, *J* = 8.6 Hz, 2'-Hb), 7.17 (1H, br t, *J* = 7.4 Hz, *p*-Ph), 7.37 (2H, br t, *J* = 8.0 Hz, *m-*Ph), 7.60 (2H, br d, *J* = 7.8 Hz, *o-*Ph), 8.49 (1H, s, 4-H). ^13^C-NMR (126 MHz, CDCl_3_): δ 13.1, 14.5, 26.1, 36.1, 39.9, 43.7, 120.5, 125.2, 127.3, 129.1, 139.0, 153.7, 166.2, 166.6, 168.9, 172.5. LC-MS: *R*t = 13.242 min, *m*/*z* = 353 (MH^+^), area% = 100. *m*/*z* (ESI) = 353 (MH^+^). *m*/*z* (HRMS) Found: 353.1973 (MH^+^). C_20_H_25_N_4_O_2_ requires: *m*/*z* = 353.1972. *ν*_max_ (KBr) 3413, 2346, 1637, 1516, 1500, 1430, 1310, 995, 761, 691 cm^−1^.

#### 3.7.9. *2-Methyl-6-(5-oxo-1-phenylpyrrolidin-3-yl)-N-(pyrrolidin-1-yl)pyrimidine-5-carboxamide* (**10**{*1*; *9*})

Prepared from **17**{*1*} and pyrrolidine (**18**{*9*}), workup B. Yield: 205 mg (79%) of yellow resin. ^1^H-NMR (500 MHz, CDCl_3_): δ 1.90–2.08 (4H, m, 4H of pyrrolidine), 2.75 (3H, s, 2-CH_3_), 2.88 (1H, dd, *J* = 9.0, 16.8 Hz, 4'-Ha), 3.17 (1H, dd, *J* = 8.7, 16.8 Hz, 4'-Hb), 3.23–3.29 (1H, m, 1H of pyrrolidine), 3.30–3.37 (1H, m, 1H of pyrrolidine), 3.68 (2H, br t, *J* = 6.9 Hz, 2H of pyrrolidine), 3.97 (1H, quintet, *J* = 8.4 Hz, 3'-H), 4.15 (1H, br t, *J* = 8.9 Hz, 2'-Ha), 4.21 (1H, dd, *J* = 7.6, 9.5 Hz, 2'-Hb), 7.17 (1H, br t, *J* = 7.4 Hz, *p-*Ph), 7.37 (2H, br t, *J* = 8.0 Hz, *m-*Ph), 7.60 (2H, d, *J* = 7.4 Hz, *o-*Ph), 8.56 (1H, s, 4-H). ^13^C-NMR (126 MHz, CDCl_3_): δ 24.6, 26.1, 26.4, 36.2, 38.3, 46.3, 49.5, 53.8, 120.6, 125.2, 127.5, 129.1, 138.9, 154.4, 165.4, 166.5, 169.1, 172.7. LC-MS: *R*t = 12.258 min, *m*/*z* = 351 (MH^+^), area% = 100. *m*/*z* (ESI) = 351 (MH^+^). *m*/*z* (HRMS) Found: 351.1815 (MH^+^). C_20_H_23_N_4_O_2_ requires: *m*/*z* = 351.1816. *ν*_max_ (KBr) 3422, 2362, 1638, 1516, 1426, 997, 764, 670 cm^−1^.

#### 3.7.10. *2-Methyl-6-(5-oxo-1-phenylpyrrolidin-3-yl)-N-(piperidin-1-yl)pyrimidine-5-carboxamide* (**10**{*1*; *10*})

Prepared from **17**{*1*} and piperidine (**18**{*10*}), workup B. Yield: 201 mg (100%) of yellow resin. ^1^H-NMR (500 MHz, CDCl_3_): δ 1.54 (2H, br s, 2H of piperidine), 1.72 (4H, br s, 4H of piperidine), 2.76 (3H, s, 2-CH_3_), 2.81–2.99 (1H, m, 4'-Ha), 3.13–3.25 (1H, m, 4'-Hb), 3.29 (2H, br s, 2H of piperidine), 3.77 (2H, br s, 2H of piperidine), 3.90 (1H, quintet, *J* = 8.5 Hz, 3'-H), 4.19 (2H, br s, 2'-CH_2_), 7.17 (1H, br t, *J* = 7.4 Hz, *p-*Ph), 7.38 (2H, br t, *J* = 8.0 Hz, *m-*Ph), 7.60 (2H, br d, *J* = 7.7 Hz, *o-*Ph), 8.47 (1H, s, 4-H). ^13^C-NMR (126 MHz, CDCl_3_): δ 24.4, 25.8, 26.1, 27.0, 36.2, 43.3, 48.9, 120.6, 125.2, 126.8, 129.1, 139.0, 154.2, 165.5, 166.4, 169.0, 172.6. LC-MS: *R*t = 12.55 min, *m*/*z* = 365 (MH^+^), area% = 100. *m*/*z* (ESI) = 365 (MH^+^). *m*/*z* (HRMS) Found: 365.197 (MH^+^). C_21_H_25_N_4_O_2_ requires: *m*/*z* = 365.1972. *ν*_max_ (KBr) 3438, 2325, 1630, 1515, 1500, 1431, 1288, 1000, 760, 692 cm^−1^.

#### 3.7.11. *2-Methyl-6-(5-oxo-1-phenylpyrrolidin-3-yl)-N-(morpholin-4-yl)pyrimidine-5-carboxamide* (**10**{*1*; *11*})

Prepared from **17**{*1*} and morpholine (**18**{*11*}), workup B. Yield: 228 mg (99%) of yellow resin. ^1^H-NMR (500 MHz, CDCl_3_): δ 2.76 (3H, s, 2-CH_3_), 2.86–2.95 (1H, m, 4'-Ha), 3.11–3.23 (1H, m, 4'-Hb), 3.32–3.43 (2H, m, 2H of morpholine), 3.60–3.71 (2H, m, 2H of morpholine), 3.79–3.88 (4H, m, 4H of morpholine), 3.91 (1H, quintet, *J* = 8.4 Hz, 3'-H), 4.12–4.25 (2H, m, 2'-CH_2_), 7.17 (1H, br t, *J* = 7.4 Hz, *o-*Ph), 7.38 (2H, br t, *J* = 8.0 Hz, *m*-Ph), 7.60 (2H, br d, *J* = 7.7 Hz, *o-*Ph), 8.48 (1H, s, 4-H). ^13^C-NMR (126 MHz, CDCl_3_): δ 26.2, 36.2, 42.7, 45.8, 48.1, 67.0, 120.6, 125.2, 125.8, 129.1, 138.9, 154.5, 165.9, 166.9, 169.5, 172.4. LC-MS: *R*t = 10.342 min, *m*/*z* = 367 (MH^+^), area% = 100. *m*/*z* (ESI) = 367 (MH^+^). *m*/*z* (HRMS) Found: 367.1766 (MH^+^). C_20_H_23_N_4_O_3_ requires: *m*/*z* = 367.1765. *ν*_max_ (KBr) 3454, 2326, 1633, 1516, 1501, 1428, 1284, 1117, 984, 840, 762, 696 cm^−1^.

#### 3.7.12. *2-Methyl-6-(5-oxo-1-phenylpyrrolidin-3-yl)-N-(4-methylpiperazin-1-yl)pyrimidine-5-carboxamide* (**10**{*1*; *12*})

Prepared from **17**{*1*} and 4-methylpiperazine (**18**{*12*}), workup B. Yield: 203 mg (100%) of yellow resin. ^1^H-NMR (500 MHz, CDCl_3_): δ 2.38 (3H, s, 4''-CH_3_), 2.39 (1H, br s, 1H of piperazine), 2.46 (1H, br s, 1H of piperazine), 2.53–2.65 (2H, m, 2H of piperazine), 2.76 (3H, s, 2-CH_3_), 2.82–2.98 (1H, m, 4'-Ha), 3.12–3.25 (1H, m, 4'-Hb), 3.40 (2H, m, 2H of piperazine), 3.78–3.87 (1H, m, 1H of piperazine), 3.89 (1H, quintet, *J* = 8.4 Hz, 3'-H), 3.90–3.97 (1H, m, 1H of piperazine), 4.17 (2H, m, 2'-CH_2_), 7.17 (1H, br t, *J* = 7.4 Hz, *p-*Ph), 7.38 (2H, br t, *J* = 8.0 Hz, *m-*Ph), 7.60 (2H, br d, *J* = 7.7 Hz, *o-*Ph), 8.47 (1H, s, 4-H). ^13^C-NMR (126 MHz, CDCl_3_): δ 26.2, 31.1, 36.2, 41.8, 45.7, 45.9, 47.3, 54.7, 55.3, 120.5, 125.2, 126.1, 129.1, 139.0, 154.5, 165.7, 166.7, 169.4, 172.4. LC-MS: *R*t = 9.683 min, *m*/*z* = 380 (MH^+^), area% = 100. *m*/*z* (ESI) = 380 (MH^+^). *m*/*z* (HRMS) Found: 380.2085 (MH^+^). C_21_H_26_N_5_O_2_ requires: *m*/*z* = 380.2081. *ν*_max_ (KBr) 3448, 2365, 1636, 1500, 1292, 1154, 986, 764, 694 cm^−1^.

#### 3.7.13. *6-(5-Oxo-1-phenylpyrrolidin-3-yl)-N-pentyl-1-phenylpyrimidine-5-carboxamide* (**10**{*2*; *1*})

Prepared from **17**{*2*} and 1-pentylamine (**18**{*1*}), workup A. Yield: 214 mg (100%) of white solid; m.p. 122–126 °C. ^1^H-NMR (500 MHz, CDCl_3_): δ 0.93 (3H, t, *J* = 7.0 Hz, CH_3_ of C_5_H_11_), 1.36–1.41 (4H, m, 2CH_2_ of C_5_H_11_), 1.62–1.68 (2H, m, CH_2_ of C_5_H_11_), 2.95 (1H, dd, *J* = 8.7, 16.9 Hz, 4'-Ha), 3.23 (1H, dd, *J* = 6.5, 16.9 Hz, 4'-Hb), 3.46 (2H, q, *J* = 6.3 Hz, CH_2_ of C_5_H_11_), 4.14 (1H, dd, *J* = 5.2, 9.1 Hz, 2'-Ha), 4.31 (1H, t, *J* = 8.6 Hz, 3'-H), 4.32–4.37 (1H, m, 2'-Hb), 6.39 (1H, s, NH), 7.14 (1H, br t, *J* = 7.4 Hz, *p-*Ph), 7.35 (2H, br t, *J* = 8.0 Hz, *m-*Ph), 7.44–7.52 (3H, m, *m,p-*Ph), 7.59 (2H, br d, *J* = 7.8 Hz, *o-*Ph), 8.44 (2H, br d, *J* = 7.0 Hz, *o-*Ph), 8.77 (1H, s, 4-H). ^13^C-NMR (126 MHz, CDCl_3_): δ 14.2, 22.5, 29.3, 29.4, 35.9, 38.3, 40.5, 54.1, 120.5, 125.0, 126.6, 128.8, 128.9, 129.0, 131.8, 136.6, 139.2, 155.4, 165.3, 165.9, 168.5, 173.1. LC-MS: *R*t = 20.192 min, *m*/*z* = 429 (MH^+^), area% = 100. *m*/*z* (ESI) = 429 (MH^+^). *m*/*z* (HRMS) Found: 429.2289 (MH^+^). C_26_H_29_N_4_O_2_ requires: *m*/*z* = 429.2285. (Found: C 72.10; H 6.44; N 12.90. C_26_H_28_N_4_O_2_ requires (428.5): C 72.87; H 6.59; N 13.07.); *ν*_max_ (KBr) 3436, 2340, 1677, 1656, 1537, 1435, 1409, 1308, 755, 693 cm^−1^.

#### 3.7.14. *N-Cyclohexyl-6-(5-oxo-1-phenylpyrrolidin-3-yl)-2-phenylpyrimidine-5-carboxamide* (**10**{*2*; *2*})

Prepared from **17**{*2*} and cyclohexylamine (**18**{*2*}), workup A. Yield: 225 mg (100%) of white solid; m.p. 196–199 °C. ^1^H-NMR (500 MHz, CDCl_3_): δ 1.21–1.32 (3H, m, 3H of C_6_H_11_), 1.40–1.48 (2H, m, 2H of C_6_H_11_), 1.65–1.71 (1H, m, 1H of C_6_H_11_), 1.76–1.82 (2H, m, 2H of C_6_H_11_), 2.03–2.10 (2H, m, 2H of C_6_H_11_), 2.95 (1H, dd, *J* = 8.8, 16.9 Hz, 4'-Ha), 3.23 (1H, dd, *J* = 6.5, 16.9 Hz, 4'-Hb), 3.96 (1H, tdd, *J* = 4.0, 8.0, 11.5 Hz, 1H of C_6_H_11_), 4.15 (1H, q, *J* = 4.8 Hz, 2'-Ha), 4.27–4–35 (2H, m, 2'-Hb, 3'-H), 6.22 (1H, d, *J* = 7.3 Hz, NH), 7.13 (1H, br t, *J* = 7.4 Hz, *p-*Ph), 7.35 (2H, br t, *J* = 8.0 Hz, *m-*Ph), 7.43–7.52 (3H, m, *p,m-*Ph), 7.59 (2H, br d, *J* = 7.8 Hz, *o-*Ph), 8.44 (2H, br d, *J* = 7.0 Hz, *o-*Ph), 8.75 (1H, s, 4-H). ^13^C-NMR (126 MHz, CDCl_3_): δ 25.1, 25.6, 33.2, 35.9, 38.2, 49.6, 54.0, 120.4, 124.9, 126.8, 128.8, 128.9, 129.0, 131.8, 136.6, 139.2, 155.4, 165.1, 165.2, 168.4, 173.0. LC-MS: *R*t = 20.275 min, *m*/*z* = 441 (MH^+^), area% = 86. *m*/*z* (ESI) = 441 (MH^+^). *m*/*z* (HRMS) Found: 441.2287 (MH^+^). C_27_H_29_N_4_O_2_ requires: *m*/*z* = 441.2285. (Found: C 73.66; H 6.13; N 12.53. C_27_H_28_N_4_O_2_ (440.5) requires: C 73.61; H 6.41; N 12.72.); *ν*_max_ (KBr) 3411, 2342, 1691, 1633, 1567, 1431, 1400, 1316, 1229, 756, 717, 693 cm^–1^.

#### 3.7.15. *N-Benzyl-6-(5-oxo-1-phenylpyrrolidin-3-yl)-2-phenylpyrimidine-5-carboxamide* (**10**{*2*; *3*})

Prepared from **17**{*2*} and benzylamine (**18**{*3*}), workup A. Yield: 173 mg (77%) of white solid; m.p. 169–171 °C. ^1^H-NMR (500 MHz, CDCl_3_): δ 2.96 (1H, dd, *J* = 8.8, 16.9 Hz, 4'-Ha), 3.26 (1H, dd, *J* = 6.7, 16.9 Hz, 4'-Hb), 4.14 (1H, dd, *J* = 5.8, 9.6 Hz, 2'-Ha), 4.31 (1H, t, *J* = 8.8 Hz, 2'-Hb), 4.39 (1H, ddd, *J* = 6.4, 8.3, 12.5 Hz, 3'-H), 4.64 and 4.68 (2H, 2dd, J = 5.7, 14.6 Hz, C*H*_2_Ph'), 6.48 (1H, t, *J* = 5.4 Hz, NH), 7.15 (1H, br t, *J* = 7.4 Hz, *p-*Ph), 7.32–7.41 (7H, m, *m*-Ph, Ph'), 7.45–7.52 (3H, m, *m,p-*Ph), 7.60 (2H, br d, *J* = 7.9 Hz, *o-*Ph), 8.46 (2H, br d, *J* = 7.0 Hz, *o-*Ph), 8.81 (1H, s, 4-H). ^13^C-NMR (126 MHz, CDCl_3_): δ 35.9, 38.2, 44.6, 54.1, 120.4, 124.9, 126.2, 128.2, 128.3, 128.9, 129.0, 129.1, 129.3, 131.9, 136.5, 137.5, 139.3, 155.4, 165.5, 165.8, 168.8, 173.0. LC-MS: *R*t = 19.4 min, *m*/*z* = 449 (MH^+^), area% = 100. *m*/*z* (ESI) = 449 (MH^+^). *m*/*z* (HRMS) Found: 449.198 (MH^+^). C_28_H_25_N_4_O_2_ requires: *m*/*z* = 449.1972. (Found: C 74.63; H 5.43; N 12.38. C_28_H_24_N_4_O_2_ (448.5) requires: C 74.98; H 5.39; N 12.49.); *ν*_max_ (KBr) 3418, 1679, 1662, 1570, 1498, 1434, 1305, 760, 692 cm^−1^.

#### 3.7.16. *N-(2-Methoxyethyl)-6-(5-oxo-1-phenylpyrrolidin-3-yl)-2-phenylpyrimidine-5-carboxamide* (**10**{*2*; *4*})

Prepared from **17**{*2*} and 2-methoxyethylamine (**18**{*4*}), workup A. Yield: 138 mg (65%) of white solid; m.p. 164–168 °C. ^1^H-NMR (500 MHz, CDCl_3_): δ 2.98 (1H, dd, *J* = 8.6, 16.8 Hz, 4'-Ha), 3.27 (1H, dd, *J* = 6.8, 16.9 Hz, 4'-Hb), 3.40 (3H, s, OCH_3_), 3.60 (2H, t, *J* = 4.9 Hz, C*H*_2_OMe), 3.66–3.69 (2H, t, *J* = 5.0 Hz, C*H*_2_NH), 4.17 (1H, dd, *J* = 5.8, 9.4 Hz, 2'-Ha), 4.32 (1H, t, *J* = 8.8 Hz, 2'-Hb), 4.34–4.40 (1H, m, 3'-H), 6.60 (1H, s, NH), 7.14 (1H, br t, *J* = 7.4 Hz, *p-*Ph), 7.36 (2H, br t, *J* = 8.0 Hz, *m-*Ph), 7.45–7.53 (3H, m, *p,m*-Ph), 7.62 (2H, br d, *J* = 7.7 Hz, *o-*Ph), 8.46 (2H, dd, *J* = 1.4, 8.1 Hz, *o-*Ph), 8.82 (1H, s, 4-H). ^13^C-NMR (126 MHz, CDCl_3_): δ 35.9, 38.3, 40.1, 54.0, 59.1, 70.9, 120.4, 124.9, 126.4, 128.8, 128.9, 129.1, 131.9, 136.6, 139.3, 155.6, 165.4, 166.0, 168.5, 173.0. LC-MS: *R*t = 15.017 min, *m*/*z* = 417 (MH^+^), area% = 100. *m*/*z* (ESI) = 417 (MH^+^). *m*/*z* (HRMS) Found: 415.1779 ([M–H]^–^). C_24_H_23_N_4_O_3_ requires: *m*/*z* = 415.1776. (Found: C 68.03; H 5.43; N 13.11. C_24_H_24_N_4_O_3_·⅖H_2_O (423.7) requires: C 68.04; H 5.90; N 13.23.); *ν*_max_ (KBr) 3466, 2934, 1682, 1663, 1568, 1432, 1306, 1122, 761, 693 cm^−1^.

#### 3.7.17. *N-(3-Hydroxypropyl)-6-(5-oxo-1-phenylpyrrolidin-3-yl)-2-phenylpyrimidine-5-carboxamide* (**10**{*2*; *5*})

Prepared from **17**{*2*} and 3-amino-1-propanol (**18**{*5*}), workup A. Yield: 206 mg (98%) of white solid; 145–146 °C. ^1^H-NMR (500 MHz, CDCl_3_): δ 1.86 (2H, quintet, *J* = 5.7 Hz, CH_2_C*H*_2_CH_2_), 2.98 (1H, dd, *J* = 8.7, 16.9 Hz, 4'-Ha), 3.21 (1H, dd, *J* = 6.5, 16.9 Hz, 4'-Hb), 3.65 (2H, br q, *J* = 6.1 Hz, C*H*_2_NH), 3.82 (2H, t, *J* = 5.5 Hz, C*H*_2_OH), 4.17 (1H, dd, *J* = 5.6, 9.6 Hz, 2'-Ha), 4.31 (1H, t, *J* = 8.9 Hz, 2'Hb), 4.38 (1H, ddd, *J* = 6.4, 8.3, 12.2 Hz, 3'-H), 7.12 (1H, br s, NH); 7.14 (1H, br t, *J* = 7.4 Hz, *p-*Ph), 7.35 (2H, br t, *J* = 8.0 Hz, *m*-Ph), 7.44–7.52 (3H, m, *m*,*p-*Ph), 7.60 (2H, br d, *J* = 7.7 Hz, *o-*Ph), 8.44 (2H, dd, *J* = 1.5, 8.5 Hz, *o-*Ph), 8.80 (1H, s, 4-H), OH exchanged. ^13^C-NMR (126 MHz, CDCl_3_): δ 31.5, 35.9, 38.4, 38.7, 54.1, 61.3, 120.6, 125.1, 126.3, 128.8, 128.9, 129.1, 131.9, 136.5, 139.2, 155.7, 165.3, 166.4, 168.6, 173.2. LC-MS: *R*t = 13.117 min, *m*/*z* = 417 (MH^+^), area% = 84. *m*/*z* (ESI) = 417 (MH^+^). *m*/*z* (HRMS) Found: 417.192 (MH^+^). C_24_H_25_N_4_O_3_ requires: *m*/*z* = 417.1921. (Found: C 68.23; H 5.60; N 13.21. C_24_H_24_N_4_O_3_·⅓H_2_O (422.5) requires: C 68.24; H 5.89; N 13.27.); *ν*_max_ (KBr) 3458, 2343, 1682, 1646, 1568, 1432, 1402, 1306, 1071, 762, 694 cm^−1^.

#### 3.7.18. *N-(3-Dimethylaminopropyl)-6-(5-oxo-1-phenylpyrrolidin-3-yl)-2-phenylpyrimidine-5-carboxamide* (**10**{*2*; *6*})

Prepared from **17**{*2*} and 3-(dimethylamino)propylamine (**18**{*6*}), workup A. Yield: 158 mg (71%) of white solid; 111–114 °C. ^1^H-NMR (500 MHz, CDCl_3_): δ 1.78–1.84 (2H, m, CH_2_C*H*_2_CH_2_), 2.29 (6H, s, NMe_2_), 2.54 (2H, t, *J* = 5.7 Hz, C*H*_2_NMe_2_), 3.00 (1H, dd, *J* = 8.9, 16.9 Hz, 4'-Ha), 3.29 (1H, dd, *J* = 6.7, 16.9 Hz, 4'-Hb), 3.54–3.65 (2H, m, C*H*_2_NH), 4.17 (1H, dd, *J* = 5.8, 9.7 Hz, 2'-Ha), 4.36 (1H, dd, *J* = 8.1, 9.7 Hz, 2'-Hb), 4.51 (1H, tt, *J* = 6.6, 8.5 Hz, 3'-H), 7.14 (1H, br t, *J* = 7.4 Hz, *p-*Ph), 7.37 (2H, br t, *J* = 8.0 Hz, *m-*Ph), 7.46–7.53 (3H, m, *p,m-*Ph), 7.64 (2H, br d, *J* = 7.7 Hz, *o-*Ph), 8.48 (2H, dd, *J* = 1.6, 7.9 Hz, *o-*Ph), 8.77 (1H, s, 4-H), 8.85 (1H, br s, NH). ^13^C-NMR (126 MHz, CDCl_3_): δ 23.7, 36.2, 38.9, 40.4, 44.6, 53.7, 58.3, 120.4, 124.8, 126.4, 128.7, 128.8, 129.0, 131.2, 137.2, 139.5, 159.6, 164.1, 168.2, 171.3, 173.8. LC-MS: *R*t = 9.342 min, *m*/*z* = 444 (MH^+^), area% = 100. *m*/*z* (ESI) = 444 (MH^+^). *m*/*z* (HRMS) Found: 444.2401 (MH^+^). C_26_H_30_N_5_O_2_ requires: *m*/*z* = 444.2394. *ν*_max_ (KBr) 3446, 2946, 1689, 1631, 1570, 1431, 754, 692 cm^−1^.

#### 3.7.19. *6-(5-Oxo-1-phenylpyrrolidin-3-yl)-2-phenyl-N-((pyridin-2-yl)methyl)pyrimidine-5-carboxamide* (**10**{*2*; *7*})

Prepared from **17**{*2*} and 2-picolylamine (**18**{*7*}), workup A. Yield: 204 mg (89%) of gray solid, m.p. 160–165 °C. ^1^H-NMR (500 MHz, CDCl_3_): δ 3.00 (1H, dd, *J* = 8.9, 16.9 Hz, 4'-Ha), 3.29 (1H, dd, *J* = 7.1, 16.9 Hz, 4'-Hb), 4.19 (1H, dd, *J* = 6.1, 9.6 Hz, 2'-Ha) 4.32 (1H, dd, *J* = 8.3, 9.6 Hz, 2'-Hb), 4.44 (1H, t, *J* = 7.0, 8.4 Hz, 3'-H), 4.76 and 4.80 (2H, 2dd, 1:1, *J* = 4.7, 17.5 Hz, C*H*_2_NH), 7.14 (1H, br t, *J* = 7.4 Hz, *p-*Ph), 7.25 (1H, br dd, *J* = 5.2, 7.1 Hz, 5''-H), 7.33–7.38 (3H, m, *p*,*m*-Ph), 7.46–7.53 (3H, m, *m-*Ph, NH), 7.62 (2H, br d, *J* = 7.7 Hz, *o-*Ph), 7.73 (2H, dt, *J* = 1.7, 7.6 Hz, 3''-H, 4''-H), 8.49 (2H, dt, *J* = 1.5, 8.1 Hz, *o-*Ph), 8.54 (1H, br d, *J* = 4.6 Hz, 6''-H), 8.96 (1H, s, 4-H). ^13^C-NMR (126 MHz, CDCl_3_): δ 35.9, 38.4, 44.7, 54.1, 120.4, 122.4, 123.0, 124.9, 126.4, 128.8, 128.9, 129.0, 131.8, 136.6, 137.3, 139.3, 149.2, 155.1, 156.0, 165.4, 165.9, 168.5, 173.0. LC-MS: *R*t = 15.65 min, *m*/*z* = 450 (MH^+^), area% = 87. *m*/*z* (ESI) = 450 (MH^+^). *m*/*z* (HRMS) Found: 448.1785 ([M−H]^−^). C_27_H_22_N_5_O_2_ requires: *m*/*z* = 448.1779. (Found: C 70.65; H 5.00; N 15.09. C_27_H_23_N_5_O_2_∙½H_2_O (458.5) requires: C 70.73; H 5.28; N 15.27.); *ν*_max_ (KBr) 3472, 1682, 1662, 1569, 1434, 1404, 1307, 758, 693 cm^−1^.

#### 3.7.20. *N*,*N-(Diethyl)-6-(5-oxo-1-phenylpyrrolidin-3-yl)-2-phenylpyrimidine-5-carboxamide* (**10**{*2*; *8*})

Prepared from **17**{*2*} and diethylamine (**18**{*8*}), workup B. Yield: 148 mg (68%) of yellowish resin. ^1^H-NMR (500 MHz, CDCl_3_): δ 1.17 and 1.32 (6H, 2t, 1:1, *J* = 7.1 Hz, 2C*H*_3_CH_2_), 2.95 (1H, dd, *J* = 8.9, 16.9 Hz, 4'-Ha), 3.24–3.34 (3H, m, 4'-Hb, C*H*_2_CH_3_), 3.60 and 3.66 (2H, 2 septets, *J* = 7.2 Hz, C*H*_2_CH_3_), 3.92 (1H, quintet, *J* = 8.0 Hz, 3'-H), 4.22 and 4.24 (2H, 2dd, 1:1, *J* = 10.0, 12.5 Hz, 2'-CH_2_), 7.16 (1H, br t, *J* = 7.4 Hz, *p-*Ph), 7.38 (2H, br t, *J* = 8.0 Hz, *m-*Ph), 7.46–7.54 (3H, m, *p*,*m*-Ph), 7.64 (2H, br d, *J* = 7.9 Hz, *o-*Ph), 8.47 (2H, dd, *J* = 1.8, 8.0 Hz, *o-*Ph), 8.65 (1H, s, 4-H). ^13^C-NMR (126 MHz, CDCl_3_): δ 11.6, 36.2, 38.8, 42.3, 53.9, 120.3, 124.8, 126.8, 128.8, 128.9, 129.1, 131.4, 137.2, 139.5, 159.8, 164.5, 168.6, 170.7, 173.6. LC-MS: *R*t = 18.008 min, *m*/*z* = 415 (MH^+^), area% = 88. *m*/*z* (ESI) = 415 (MH^+^). *m*/*z* (HRMS) Found: 415.2121 (MH^+^). C_25_H_27_N_4_O_2_ requires: *m*/*z* = 415.2129. (Found: C 69.44; H 6.41; N 12.62. C_25_H_26_N_4_O_2_·H_2_O (432.5) requires: C 69.42; H 6.53; N 12.95.); *ν*_max_ (KBr) 3410, 2364, 1665, 1638, 1616, 1500, 1393, 1366, 1312, 751, 717, 690 cm^−1^. 

#### 3.7.21. *6-(5-Oxo-1-phenylpyrrolidin-3-yl)-2-phenyl-N-(pyrrolidin-1-yl)pyrimidine-5-carboxamide* (**10**{*2*; *9*})

Prepared from **17**{*2*} and pyrrolidine (**18**{*9*}), workup B. Yield: 208 mg (100%) of yellow resin. ^1^H-NMR (500 MHz, CDCl_3_): δ 1.93–2.10 (4H, m, 4H of pyrrolidine), 2.97 (2H, dd, *J* = 8.9, 16.9 Hz, 4'-Ha), 3.28 (1H, dd, *J* = 7.8, 16.9 Hz, 4'-Hb), 3.29–3.36 and 3.37–3.43 (2H, 2m, 1:1, 2H of pyrrolidine), 3.72 (2H, t, *J* = 7.0 Hz, 2H of pyrrolidine), 4.06 (1H, quintet, *J* = 8.1 Hz, 3'-H), 4.24 (1H, dd, *J* = 6.8, 9.6 Hz, 2'-Ha), 4.27 (1H, dd, *J* = 8.2, 9.7 Hz, 2'-Hb), 7.16 (1H, br t, *J* = 7.4 Hz, *p-*Ph), 7.38 (2H, br t, *J* = 8.0 Hz, *m-*Ph), 7.45–7.53 (3H, m, *m,p-*Ph), 7.63 (2H, br d, *J* = 7.7 Hz, *o-*Ph), 8.47 (2H, dd, *J* = 1.8, 8.2 Hz, *o-*Ph), 8.72 (1H, s, 4-H). ^13^C-NMR (126 MHz, CDCl_3_): δ 24.6, 26.4, 36.3, 38.3, 46.3, 49.5, 53.9, 120.4, 120.5, 125.1, 127.8, 128.7, 128.9, 129.1, 131.7, 136.7, 139.2, 155.2, 159.6, 164.8, 172.7. LC-MS: *R*t = 17.075 min, *m*/*z* = 413 (MH^+^), area% = 100. *m*/*z* (ESI) = 413 (MH^+^). *m*/*z* (HRMS) Found: 413.1975 (MH^+^). C_25_H_25_N_4_O_2_ requires: *m*/*z* = 413.1972. *ν*_max_ (KBr) 3431, 2361, 1636, 1500, 1418, 983, 754, 704, 668 cm^−1^.

#### 3.7.22. *6-(5-Oxo-1-phenylpyrrolidin-3-yl)-2-phenyl-N-(piperidin-1-yl)pyrimidine-5-carboxamide* (**10**{*2*; *10*})

Prepared from **17**{*2*} and piperidine (**18**{*10*}), workup B. Yield: 214 mg (100%) of yellow resin. ^1^H-NMR (500 MHz, CDCl_3_): δ 1.51–1.61 (2H, m, 2H of piperidine), 1.74 (4H, br s, 4H of piperidine), 2.96 (1H, br s, 4'-Ha), 3.27 (1H, br s, 4'-Hb), 3.34 (2H, br s, 2H of piperidine), 3.79 (2H, br s, 2H of piperidine), 3.98 (1H, quintet, *J* = 7.9 Hz, 3'-H), 4.24 (2H, br s, 2'-CH_2_), 7.16 (1H, br t, *J* = 7.4 Hz, *p-*Ph), 7.38 (2H, br t, *J* = 7.9 Hz, *m*-Ph), 7.47–7.54 (3H, m, *m,p-*Ph), 7.64 (2H, br d, *J* = 7.8 Hz, *o-*Ph), 8.47 (2H, dd, *J* = 1.9, 7.9 Hz, *o-*Ph), 8.63 (1H, s, 4-H). ^13^C-NMR (126 MHz, CDCl_3_): δ 22.6, 22.9, 36.0, 38.6, 44.6, 54.0, 120.4, 124.7, 127.0, 128.6, 128.7, 129.0, 131.2, 137.2, 139.4, 159.6, 164.2, 168.4, 170.9, 173.8. LC-MS: *R*t = 18.608 min, *m*/*z* = 427 (MH^+^), area% = 100. *m*/*z* (ESI) = 427 (MH^+^). *m*/*z* (HRMS) Found: 427.2135 (MH^+^). C_26_H_27_N_4_O_2_ requires: *m*/*z* = 427.2129. *ν*_max_ (KBr) 3438, 2326, 1630, 1515, 1500, 1431, 1288, 1000, 760, 692 cm^−1^.

#### 3.7.23. *6-(5-Oxo-1-phenylpyrrolidin-3-yl)-N-(morhpolin-4-yl)-2-phenylpyrimidine-5-carboxamide* (**10**{*2*; *11*})

Prepared from **17**{*2*} and morpholine (**18**{*11*}), workup B. Yield: 204 mg (95%) of yellow resin. ^1^H-NMR (500 MHz, CDCl_3_): δ 2.97 (1H, dd, *J* = 8.3, 16.3 Hz, 4'-Ha), 3.27 (1H, br dd, *J* = 7.0, 16.3 Hz, 4'-Hb), 3.38–3.49 (2H, m, 2H of morpholine), 3.63–3.72 (2H, m, 2H of morpholine), 3.80–3.92 (4H, m, 4H of morpholine), 3.99 (1H, quintet, *J* = 7.7 Hz, 3'-H), 4.23 and 4.25 (2H, 2br d, 1:1, 2'-CH_2_), 7.17 (1H, br t, *J* = 7.4 Hz, *p-*Ph), 7.38 (2H, br t, *J* = 8.0 Hz, *m*-Ph), 7.47–7.55 (3H, m, *m*,*p-*Ph), 7.64 (2H, br d, *J* = 7.7 Hz, *o-*Ph), 8.47 (2H, dd, *J* = 1.5, 8.0 Hz, *o-*Ph), 8.64 (1H, s, 4-H). ^13^C-NMR (126 MHz, CDCl_3_): δ 35.9, 38.6, 43.3, 54.0, 64.2, 120.4, 124.8, 126.7, 128.6, 128.7, 129.0, 131.2, 137.1, 139.3, 159.7, 164.2, 168.5, 170.8, 173.8. LC-MS: *R*t = 16.042 min, *m*/*z* = 429 (MH^+^), area% = 100. *m*/*z* (ESI) = 429 (MH^+^). *m*/*z* (HRMS) Found: 429.1921 (MH^+^). C_25_H_25_N_4_O_3_ requires: *m*/*z* = 429.1921. *ν*_max_ (KBr) 3449, 2366, 1669, 1607, 1500, 1368, 1313, 1125, 752, 717,689 cm^−1^.

#### 3.7.24. *6-(5-Oxo-1-phenylpyrrolidin-3-yl)-N-(4-methylpiperazin-1-yl)-2-phenylpyrimidine-5-carboxamide* (**10**{*2*; *12*})

Prepared from **17**{*2*} and 4-methylpiperazine (**18**{*12*}), workup B. Yield: 225 mg (100%) of yellow resin. ^1^H-NMR (500 MHz, CDCl_3_): δ 2.37 (3H, s, 4''-CH_3_), 2.36–2.42 (1H, m, 1H of piperazine), 2.46 (1H, br s, 1H of piperazine), 2.53–2.64 (2H, m, 2H of piperazine), 2.92–3.03 (1H, m, 4'-Ha), 3.24–3.34 (1H, m, 4'-Hb), 3.45 (2H, br s, 2H of piperazine), 3.85 (1H, br s, 1H of piperazine), 3.95 (1H, br s, 1H of piperazine), 3.98 (1H, quintet, *J* = 7.9 Hz, 3'-H), 4.25 (2H, br s, 2'-CH_2_), 7.17 (1H, br t, *J* = 7.4 Hz, *p-*Ph), 7.38 (2H, br t, *J* = 8.0 Hz, *m-*Ph), 7.46–7.54 (3H, m, *m,p-*Ph), 7.63 (2H, d, *J* = 7.8 Hz, *o-*Ph), 8.47 (2H, dd, *J* = 1.5, 8.0 Hz, *o-*Ph), 8.64 (1H, s, 4-H). ^13^C-NMR (126 MHz, CDCl_3_): δ 36.3, 42.0, 46.1, 47.5, 54.8, 55.5, 120.5, 125.1, 126.4, 128.7, 128.9, 129.1, 131.8, 136.6, 139.1, 155.2, 165.0, 165.8, 166.9, 172.5. LC-MS: *R*t = 9.392 min, *m*/*z* = 442 (MH^+^), area% = 100. *m*/*z* (ESI) = 380 (MH^+^). *m*/*z* (HRMS) Found: 442.2238 (MH^+^). C_26_H_28_N_5_O_2_ requires: *m*/*z* = 442.2238. *ν*_max_ (KBr) 3456, 2340, 1637, 1500, 1421, 1298, 1168, 983, 760, 696 cm^−1^.

## 4. Conclusions

2-Substituted 6-(5-oxo-1-phenylpyrrolidin-3-yl)pyrimidine-5-carboxamides **10** as a novel type of conformationally constrained 2-(heteroaryl)ethylamines are available in six-steps from itaconic acid (**11**). The synthetic pathway consists of two parts: (a) a five-step preparation of pyrimidine-5-carboxylic acids **17**{*1*,*2*} as the key-intermediates and (b) combinatorial solution-phase BPC-mediated amidation of **17**{*1*,*2*} with primary and secondary amines **18**{*1*–*12*} to give the title compounds **10**{*1*,*2*; *1*–*12*} in good overall yields and purity upon simple workup. The method is general and substrate-independent. All 24 amidations proceeded smoothly and no major differences in reactivity was observed with respect to the C(2) substituent in the pyrimidine-5-carboxylic acids **17**. On the other hand, the secondary amines **18**{*8*–*12*} were less reactive in these amidations than the primary amines **18**{*1*–hy,lip98u*7*}. Consequently, a 10-fold excess of secondary amines **18**{*8*–*12*} was employed in order to assure completion of the amidation reaction. Besides, preparation of the 2-chloro analogue of **16**, e.g*.*, by treatment of **14** with methyl carbamimidate followed by demethylation and chlorination, would enable functionalization at position 2 in the pyrimidine ring, either by S_N_Ar reaction, or by cross-coupling reaction. These results also indicate that the above synthetic method could serve as a useful tool for the preparation of novel compound libraries for pharmaceutical and other practical applications.
